# Anti-Retroviral Therapy Decreases but Does Not Normalize Indoleamine 2,3-Dioxygenase Activity in HIV-Infected Patients

**DOI:** 10.1371/journal.pone.0100446

**Published:** 2014-07-01

**Authors:** Jun Chen, Jiasheng Shao, Rentian Cai, Yinzhong Shen, Renfang Zhang, Li Liu, Tangkai Qi, Hongzhou Lu

**Affiliations:** 1 Department of Infectious Diseases, Shanghai Public Health Clinical Center, Fudan University, Shanghai, China; 2 Department of Infectious Diseases, HuaShan Hospital, Fudan University, Shanghai, China; 3 Department of Internal Medicine, Shanghai Medical College, Fudan University, Shanghai, China; University Hospital of Heidelberg, Germany

## Abstract

**Background:**

Indoleamine 2,3-dioxygenase (IDO), which is mainly expressed in activated dendritic cells, catabolizes tryptophan to kynurenine and other downstream catabolites. It is known to be an immune mediator in HIV pathogenesis. The impact of anti-retroviral therapy on its activity has not been well established.

**Methods:**

We measured systemic IDO activity (the ratio of plasma kynurenine to tryptophan) in HIV-infected patients before and after highly active antiretroviral therapy (HAART) and its association with a microbial translocation marker, soluble CD14 (sCD14).

**Results:**

Among 76 participants, higher baseline IDO activity was associated with lower CD4^+^ T cell counts (*P*<0.05) and higher plasma sCD14 levels (*P*<0.001). After 1 year of HAART, IDO activity decreased significantly (*P*<0.01), but was still higher than in healthy controls (*P*<0.05). The baseline IDO activity did not predict CD4^+^ T cell recovery after 1 year of therapy. The percentages of myeloid and plasmacytoid dendritic cells were not correlated with IDO activity.

**Conclusions:**

IDO activity is elevated in HIV-infected patients, which is partially associated with microbial translocation. HAART reduced, but did not normalize the activity of IDO.

## Introduction

Tryptophan (Trp) is an essential amino acid that is important for protein synthesis and the biosynthesis of the neurotransmitter 5-hydroxytryptamine. Indoleamine 2,3-dioxygenase (IDO) is an intracellular enzyme that catalyzes the initial rate-limiting step in Trp catabolism, leading to the production of immunoregulatory catabolites (collectively known as kynurenines [Kyn]) [1]. IDO was initially identified as part of the initial host defense response against infectious pathogens and several studies have documented its ability to inhibit the proliferation of intracellular pathogens *in vitro* by starving Trp [2], [3].

However, IDO has now been deemed as an immune mediator in HIV pathogenesis. The IDO gene promoter contains multiple sequence elements that are responsive to proinflammatory mediators [4]. Chronic HIV-1 infection is characterized by persistent microbial translocation and immune activation, suggesting that IDO activity might be increased during HIV infection. Indeed, HIV-induced IDO expression in activated dendritic cells (DCs) catabolizes Trp to Kyn, which inhibits T cell proliferation and interleukin (IL)-17 production [5]–[7]. Additionally, HIV can induce IDO-competent DCs to generate regulatory T (Treg) cells that suppress anti-HIV immune responses [8].

In the pre-antiretroviral therapy era, high IDO activity had been documented in untreated HIV infection and associated with disease progression [9]–[11]. High IDO activity also predicts earlier mortality in elderly HIV-uninfected individuals [12], [13]. So far, however, few studies have addressed the impact of highly active antiretroviral therapy (HAART) on IDO activity in HIV-infected patients [9], [10], [14]. In this study, we measured plasma levels of Trp and Kyn in a HIV-infected cohort initiating HAART and assessed its relationship with microbial translocation.

## Methods

### Study population

HIV-infected patients naïve to therapy (n = 76) and healthy controls (n = 16) were enrolled at Shanghai Public Health Clinical Center. HIV-infected patients with opportunistic infections were excluded in order to avoid the influence of other pathogens. Ethical approval was granted by the Ethics Committee of Shanghai Public Health Clinical Center. Written informed consent was obtained from each subject. Clinical data and blood samples from patients were collected. Whole blood samples (5 mL) were obtained from all subjects and plasma samples were collected for the quantification of Trp and Kyn concentrations, as well as soluble CD14 (sCD14) levels. An additional 5 mL whole blood sample was obtained from a random subset of patients naïve to therapy (n = 21) and from all healthy controls. Peripheral blood mononuclear cells (PBMC) were isolated and stained for myeloid DC (mDC) and plasmacytoid DC (pDC) markers using the following methods.

### The measurement of plasma concentrations of tryptophan and kynurenine, and IDO activity

Plasma samples were heat-inactivated and stored at −80°C before they were analyzed. Plasma concentrations of Trp and Kyn were simultaneously determined using high-performance liquid chromatography, as previously described [15]. IDO was measured as the kynurenine/tryptophan ratio (K/T ratio) and expressed in nM/µM units.

### Quantification of plasma sCD14 levels

Plasma samples were diluted and sCD14 was measured in duplicate using a commercially available ELISA assay (R&D Systems, Minneapolis, MN, USA), and analyzed according to the manufacturer's recommended procedure.

### Flow cytometry

Cell surface markers were measured using a flow cytometer (BD Biosciences, San Jose, CA, USA). The following antibodies were used: Linage-FITC, HLA-DR-APC, CD123-PE, and CD11c-PerCP-Cy5.5 (Biolegend, San Diego, CA, USA). The Fixable Viability Dye eFluor 780 (eBiosciense, San Diego, CA, USA) was used to exclude dead cells from the analysis. Magnetic beads coated with anti-rat and -mouse antibodies were used as positive and/or negative controls for calculating compensation (BD Biosciences, San Jose, CA, USA). We characterized mDC as Linage^−^HLA-DR^+^CD11c^+^, while pDC were characterized as Linage^−^HLA-DR^+^CD123^+^. Data was exported and analyzed using FlowJo software v7.6.1.

### Statistical analysis

The normality of the data was assessed using the Shapiro–Wilk test. Normally distributed data were shown as the mean ± standard deviation, whereas non-normally distributed data were shown as the median (with the interquartile range). Student's *t*-test was performed to assess differences between two groups. Wilcoxon rank-sum tests were used for non-normally distributed data. Matched samples were analyzed using paired Student's *t*-test. For correlation calculations, Spearman's rank correlation coefficient “rho” was used. Analyses were two-sided and the confidence intervals (CI) were set to 95%. Results were considered to be significant when the p-value was less than 0.05. All statistical analyses were done using STATA 12.0 (StataCorp, College Station, TX, USA) and GraphPad Prism 6.0 software (GraphPad Software Inc., San Diego, CA, USA).

## Results

### The characteristics of study populations

Among the 76 enrolled HAART-naïve patients, 76.3% (58/76) were male with a median age of 32 (28–39.5) years old. These characters were comparable to the healthy control cohort (Table 1). The baseline CD4^+^ T cell count of enrolled HIV-infected patients was 248 cells/mm^3^ (121–297 cells/mm^3^). The majority of patients (96.1%, 73/76) received two nucleoside reverse-transcriptase inhibitors (NRTIs) plus one non-nucleoside reverse-transcriptase inhibitor (NNRTIs), while the other 3.9% (3/76) of patients received two NNRITs plus one protease inhibitor (PI) regimens. The viral load of most patients (94.7%, 72/76) was undetectable (<40 copies/mL) after 1 year of therapy.

**Table 1:** Clinical characteristics of study groups.

Characteristics	HIV-infected patients (n = 76)	Healthy controls (n = 16)
Age [median (IQR)]	32 (28–39.5)	32 (27–47)
Male [n (%)]	58 (76.3%)	10% (62.5%)
CD4^+^ T cell count at baseline (cells/mm^3^) median (IQR)	248 (121–297).	N/A
CD8^+^ T cell count at baseline (cells/mm^3^) median (IQR)	620 (443–917)	N/A
CD4 to CD8 ratio, median (IQR)	0.32 (0.18–0.46)	N/A
HAART regimens		
2 NRTIs+1 NNRTIs [n (%)]	73 (96.1%)	NA
2 NRTIs+1 PIs [n (%)]	3 (3.9%)	NA
Undetectable viral load after 12 months of HAART [n (%)]	72 (94.7%)	NA

IQR, interquartile range; NRTIs, nucleoside reverse transcriptase inhibitors; NNRTIs, non-nucleoside reverse transcriptase inhibitors; PIs, protease inhibitors; N/A, not available; NA, not applicable.

### HAART could normalize plasma Trp and Kyn concentrations, but not IDO activity

**Figure 1:**
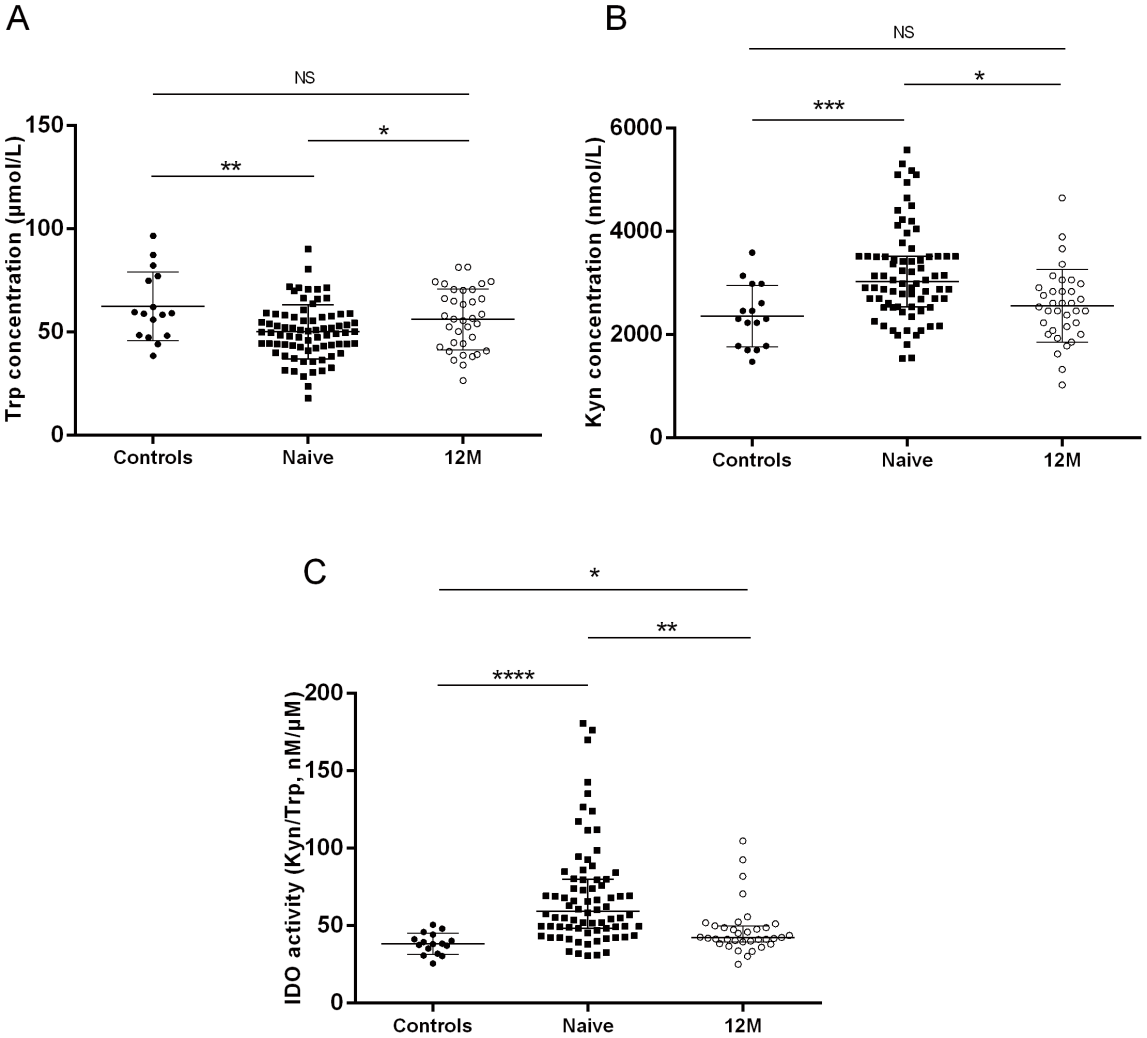
The impact of HAART on plasma concentrations of tryptophan (A) and kynurenine (B) and the activity of IDO (C) in HIV-infected patients. NS, not significant; *, *P*<0.05; **, *P*<0.01; ***, *P*<0.001; ****, *P*<0.0001.

Plasma concentrations of Trp were lower in HIV-infected patients naïve to therapy, while the plasma concentrations of Kyn were significantly higher when compared to healthy controls (62.38±4.17 µM vs. 50.04±1.50 µM; *P* = 0.0015; and 3022 nM [2531–3513 nM] vs. 2304 nM [1775–2795 nM] for Trp and Kyn, respectively; *P* = 0.0005; Figure 1A, 1B). After 12 months of therapy, both Trp and Kyn levels changed significantly and became comparable to healthy controls (Figure 1A, 1B). The IDO activities, indicated by the Kyn/Trp ratio, were also elevated in HAART-naïve patients (59.43 nM/µM [48.27–79.82 nM/µM] vs. 38.25 nM/µM [33.56–42.79 nM/µM]; *P*<0.0001; Figure 1C). One year of HAART significantly reduces IDO activities (59.43 nM/µM [48.27–79.82 nM/µM] vs. 42.43 nM/µM [39.63–49.97] pre- and post-HAART, respectively; *P* = 0.0025), but still could not normalize it (42.43 nM/µM [39.63–49.97] vs. 38.25 nM/µM [33.56–42.79 nM/µM]; *P* = 0.0148; Figure 1C).

### IDO activities were reversely correlated with baseline CD4^+^ T cell counts

**Figure 2:**
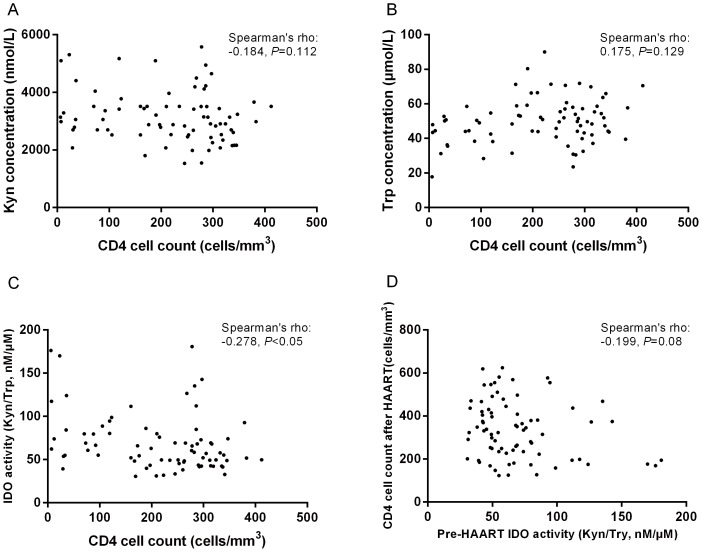
The relationship between CD4^+^ T cell counts and plasma concentrations of tryptophan (A) and kynurenine (B), and the activity of IDO (C) in HIV-infected patients. The relationship between baseline IDO activity and CD4^+^ T cell counts after one year of therapy (D).

The IDO activities in HIV patients, but not plasma concentrations of Trp or Kyn, were inversely correlated to CD4^+^ T cell counts in HIV-infected patients naïve to therapy (Figure 2A–C). IDO activities and plasma concentrations of Kyn were also correlated with the CD4 to CD8 ratio (Figure S1).However, these correlations were lost among patients after 12 months of therapy (data not shown). Meanwhile, neither the baseline IDO levels nor the concentrations of Trp or Kyn were correlated with the CD4^+^ T cell counts of patients after 12 months of therapy (Figure 2D). The delta changes of CD4 T cells counts were also not correlated with baseline IDO activity (Figure S2A).

### IDO activities were correlated with sCD14 in HIV-infected patients naïve to therapy

**Figure 3:**
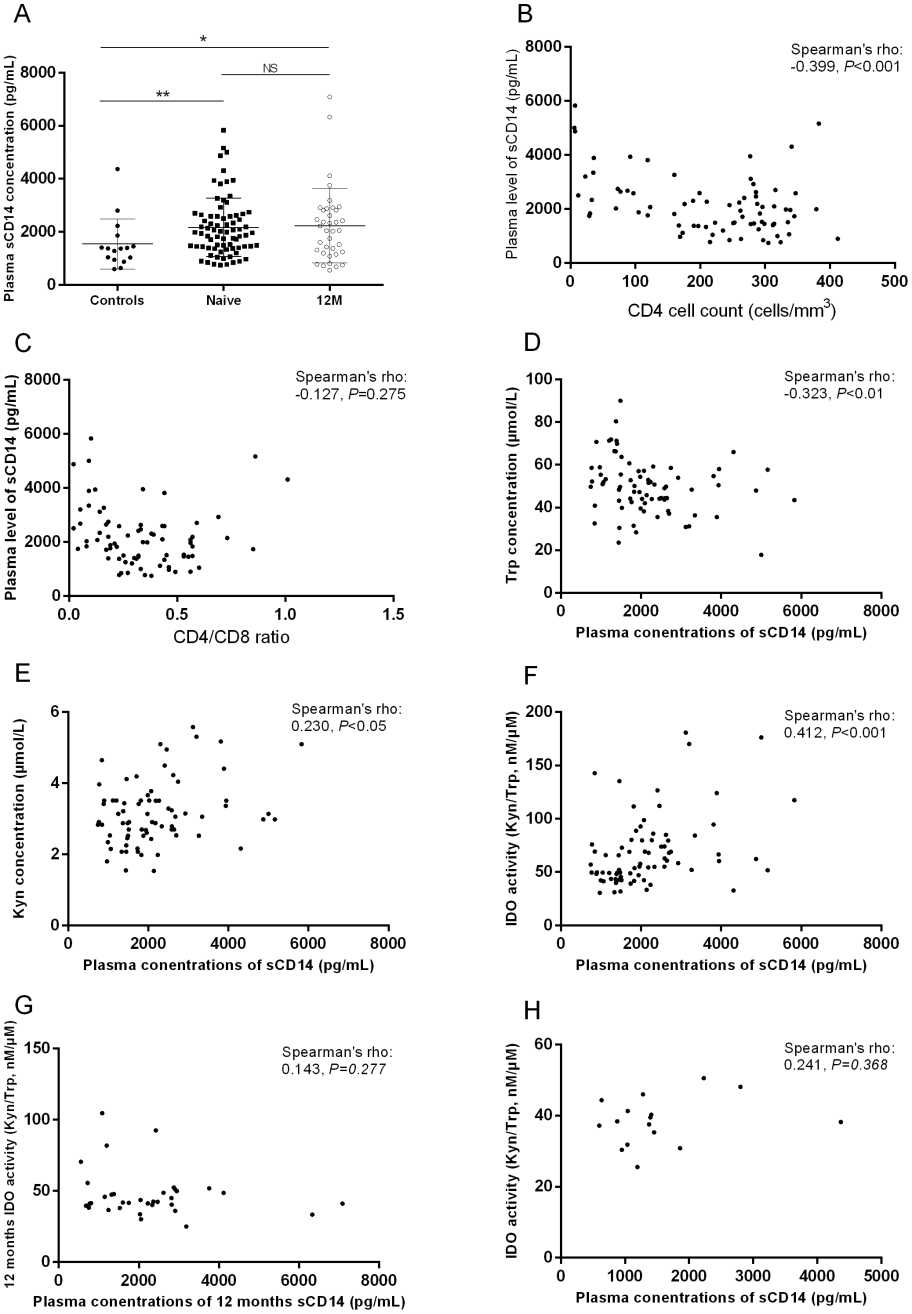
The relationship between plasma sCD14 levels and the kynurenine pathway of tryptophan catabolism. The impact of HAART on the plasma concentrations of sCD14, including the relationship with the following: CD4^+^ T cell counts (A), CD4 to CD8 ratio (B), and plasma levels of sCD14 (C). The relationship between plasma sCD14 levels with tryptophan (D) and kynurenine (E) levels and the activity of IDO (F) in HIV-infected patients naïve to therapy. The correlation of IDO activity with sCD14 levels in patients after therapy (G) and in healthy controls (H).

Plasma sCD14 levels were significantly higher among HIV-infected patients naïve to therapy when compared to healthy controls (1971 pg/mL [1422–2608 pg/mL] vs. 1372 pg/mL [994–1657 pg/mL]; *P* = 0.0076; Figure 3A). It was negatively correlated with CD4^+^ T cell counts, but not with the baseline CD4 to CD8 ratio (Figure 3B, 3C). Plasma sCD14 levels could not predict the delta changes of CD4 T cell counts after 1 year of therapy (Figure S2B). HAART did not significantly change its concentration (1971 pg/mL [1422–2608 pg/mL] vs. 2054 pg/mL [1243–2820 pg/mL] for pre- and post-HAART, respectively; *P* = 0.4492; Figure 3A). It was positively correlated with plasma concentrations of Kyn and IDO activities, while it was reversely correlated to Trp concentrations among patients naïve to therapy (Figure 3D–F). However, we did not find any correlation between plasma sCD14 levels and Kyn, Trp, or IDO in healthy controls or in patients who received 1 year of therapy (Figure 3G, 3H).

### DC percentages were not correlated with IDO activity

**Figure 4:**
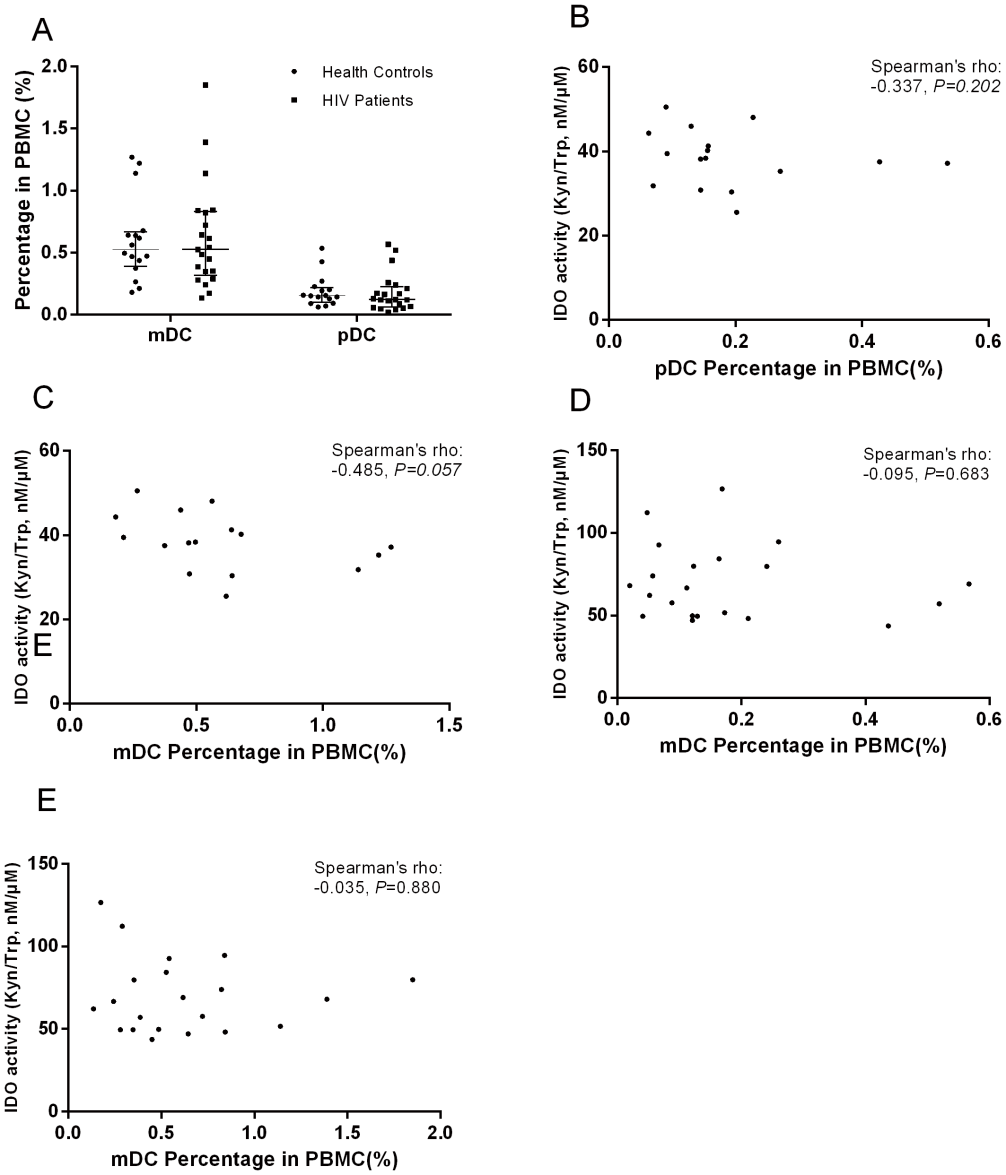
The relationship between the percentages of DCs in PBMC with IDO activity. (A) A comparison of the percentage of DCs in HIV-infected patients and healthy controls. The relationship between the percentage of pDCs (B) and mDCs (C) in PBMC with IDO activity in healthy controls and in HIV-infected patients (D and E).

DCs—the main source of IDO production—are dysregulated in HIV infection. We investigated whether the percentage of DCs in peripheral blood was correlated with IDO activity. The percentages of both mDC and pDC in PBMC among patients naïve to therapy were similar to those among healthy controls (0.529% [0.407–0.659%] vs. 0.526% [0.347–0.824%]; *P* = 0.9145; and (0.155% [0.111–0.428%] vs. 0.123% [0.048–0.211%] for mDC and pDC, respectively; *P* = 0.3419; Figure 4A.) The mDC and pDC percentages were not correlated with IDO activity in HIV patients (Figure 4B, 4C) or healthy controls (Figure 4D, 4E). The mDC and pDC percentages also did not correlate with plasma concentrations of Kyn or Trp (data not shown).

## Discussion

While the kynurenine pathway of tryptophan catabolism has been associated with immunosuppression and mental disorders in HIV infection, few studies have assessed the changes in this pathway during treatments of HIV infections [10], [14], [16]. In this study, we showed that the IDO activity in HIV-infected patients naïve to therapy was higher than in healthy controls, and HAART could decrease IDO activity, but not normalize it. We also confirmed that the increased IDO activity is partially associated with microbial translocation.

Similar to previous studies, we found that IDO activity and Kyn concentrations in HIV-infected patients were elevated, while Trp concentrations were decreased, indicating that tryptophan was more likely to be degraded through the kynurenine pathway in this population [10], [14]. Consistent with previous studies, we also found that the baseline IDO activity was inversely correlated with the baseline CD4^+^ T cell count in HIV-infected patients naïve to therapy [14], [17]. Increased IDO activity could partially lead to CD4^+^ T cell depletion. The tryptophan catabolites kynurenine and picolinic acid can inhibit T cell proliferation [18], [19]. However, it is also possible that the elevation of IDO activity and depletion of CD4^+^ T cells are both phenomena caused by HIV itself, as the correlation between CD4^+^ T cell counts and IDO activity in HIV-infected patients was abolished after effective therapy. Although the baseline IDO activity was not correlated with CD4^+^ T cell recovery after therapy, one recent study did find that high IDO activity independently predicts poor CD4^+^ T cell recovery and increased mortality among HIV-infected Ugandans initiating ART, indicating its significant prognostic value [14]. IDO activity was also correlated with disease progression [9], and it has been reported to be higher in HIV progressors than in elite controllers [17]. Besides immunological effects, IDO may have important psychological effects [20]–[22]. Some downstream tryptophan catabolites mediated by IDO are neurotoxic and might contribute to cognitive impairment or mood disorders [23]. Another study from Uganda showed that depression symptoms among HIV-infected individuals could be partially attributed to IDO-mediated tryptophan catabolism and that HAART could partially improve depression [24]. Because IDO activity in HIV-infected patients was not normalized in our patient cohort, even after 1 year of therapy, additional therapeutic methods should be investigated to reduce IDO activities in the future.

We found that plasma sCD14 levels were positively correlated with IDO activity in HIV-infected patients naïve to therapy. sCD14 is a marker of microbial translocation across the intestinal mucosa [25]–[27], which is also independently predictive of virologic response of HAART, disease progression, and HIV mortality [28],[29]. One previous study showed that the tryptophan catabolite 3-hydroxyanthralinic acid (HAA) could induce the loss of Th17 cells in HIV-infected individuals [30]. Taking into account the important role of Th17 cells in protecting against pathogens in the gut, this finding could partially explain our result. However, it is also possible that the increased IDO activity was induced by microbial products that translocated from the leaky gut in HIV-infected patients, as many microbes and microbial components were shown to be capable of inducing IDO [31]–[34]. In agreement with previous studies, the plasma sCD14 levels did not change significantly despite 1 year of effective antiretroviral therapy in our study, suggesting that HAART may not restore the intestinal barrier function, resulting in persistent microbial translocation and immune activation [25], [27], [35], [36]. Interestingly, IDO activity was also not normalized after 1 year of therapy, indicating that another factor distinct from HIV (e.g. microbes and microbial products) also triggered IDO expression, although plasma sCD14 levels did not correlate with IDO activities. In a recent study, gut-resident bacteria with the capacity to catabolize tryptophan through the kynurenine pathway were found to be enriched and strongly correlated with kynurenine levels in HIV-infected subjects, and were capable of inducing kynurenine production in vitro [37]. Thus, further research to investigate the role of microbial translocation in IDO production in HIV-infected patients is warranted. In addition, mDCs and pDCs could also be identified using other markers (e.g. CD1a and CD1c for mDCs, BDCA-2 for pDCs) which we didn't select. Further studies using these new markers to identify DCs and explore the correlation between IDO expression and DCs in HIV infection will be needed.

It is known that HIV can modulate pDCs activation through Toll-like receptor 7 stimulation, resulting in increased IDO expression [8], [38], [39]. It has also been reported that the persistence of elevated IDO activity is derived from mDCs [30], [40]. However, the findings of this current study do not support the previous findings. We found that neither the percentage of mDCs nor pDCs was correlated with IDO activity. There are several possible explanations for this result. It is possible that our results were different because we measured total mDCs and pDCs, but not IDO-expressing DCs, and we determined DC percentages, but not absolute cell counts. It is also possible that both pDCs and mDCs are the source of IDO in HIV-infected patients [30].

There are some limitations to our study. First, the HIV viral load was reported to be positively correlated with IDO activities, and this was not detected at baseline levels in this study. Second, the K/T ratio is not specific for IDO activity. Besides IDO and enzymes produced by gut-resident bacteria, it could also be affected by tryptophan 2,3-dioxygenase (TDO), which is not inducible and is mainly expressed in the liver [41]–[43]. Thus, the relatively high K/T ratio found in HIV infected patients is not likely to be driven by IDO expression.

Collectively, our results show that IDO activity is elevated in HIV-infected patients, which was partially associated with microbial translocation. HAART could decrease IDO activity, but not fully corrected it after one year treatment.

## Supporting Information

Figure S1 The relationship between CD4 to CD8 ratio and plasma concentrations of tryptophan (A) and kynurenine (B), and the activity of IDO (C) in HIV-infected patients.
(TIF)

Figure S2 The relationship between delta CD4^+^ T cell changes from baseline to IDO activity (A) and plasma sCD14 level (B) in HIV-infected patients.
(TIF)

Raw Data S1 (ZIP)
