# Supplementary figures and images for: Anti-Retroviral Therapy Decreases but Does Not Normalize Indoleamine 2,3-Dioxygenase Activity in HIV-Infected Patients
Source: PLoS One. 2014 Jul 1;9(7):e100446. doi: 10.1371/journal.pone.0100446 (PMC4077698; doi:10.1371/journal.pone.0100446)

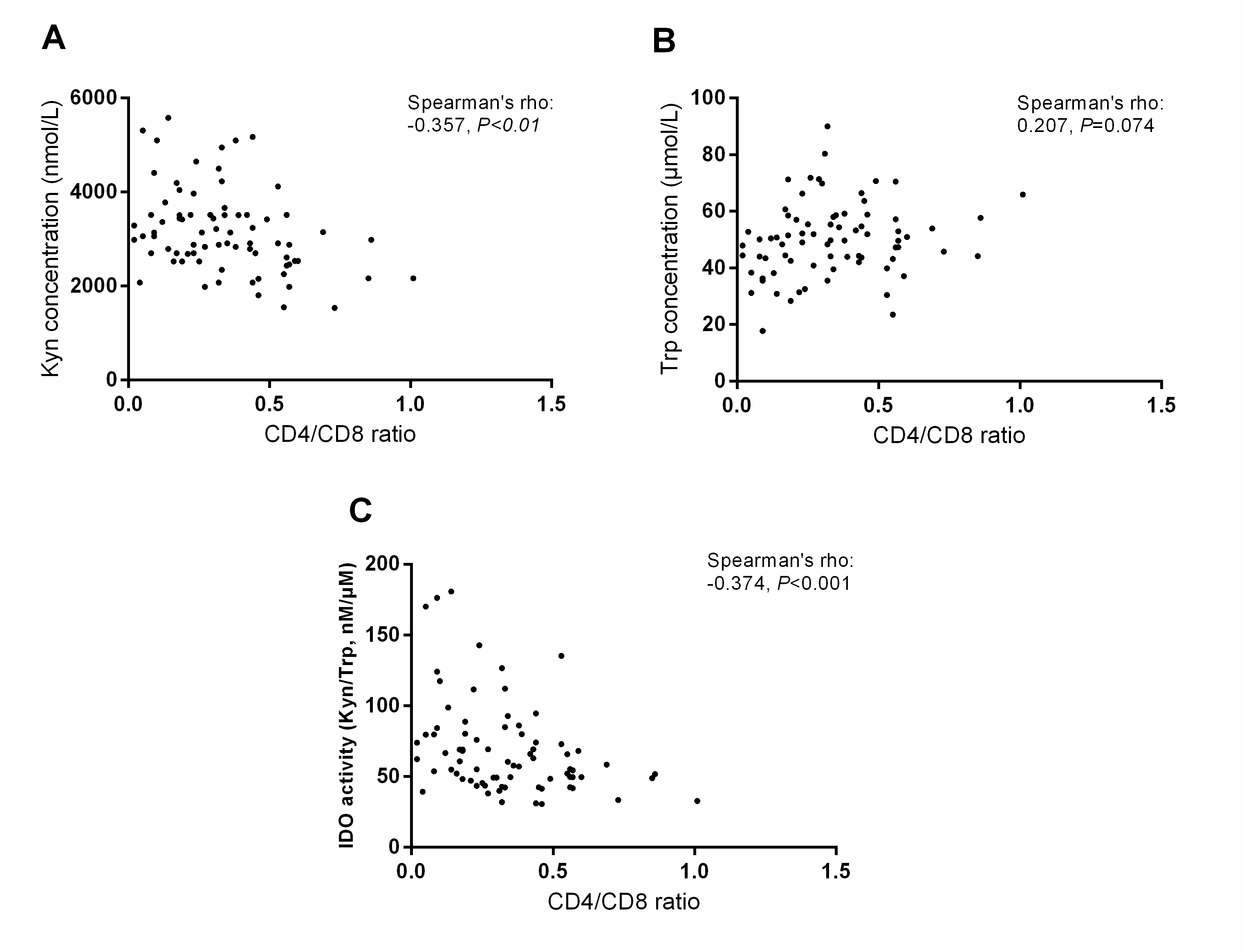

Supplement: Figure S1 — The relationship between CD4 to CD8 ratio and plasma concentrations of tryptophan (A) and kynurenine (B), and the activity of IDO (C) in HIV-infected patients. (TIF) [file pone.0100446.s001.tif]

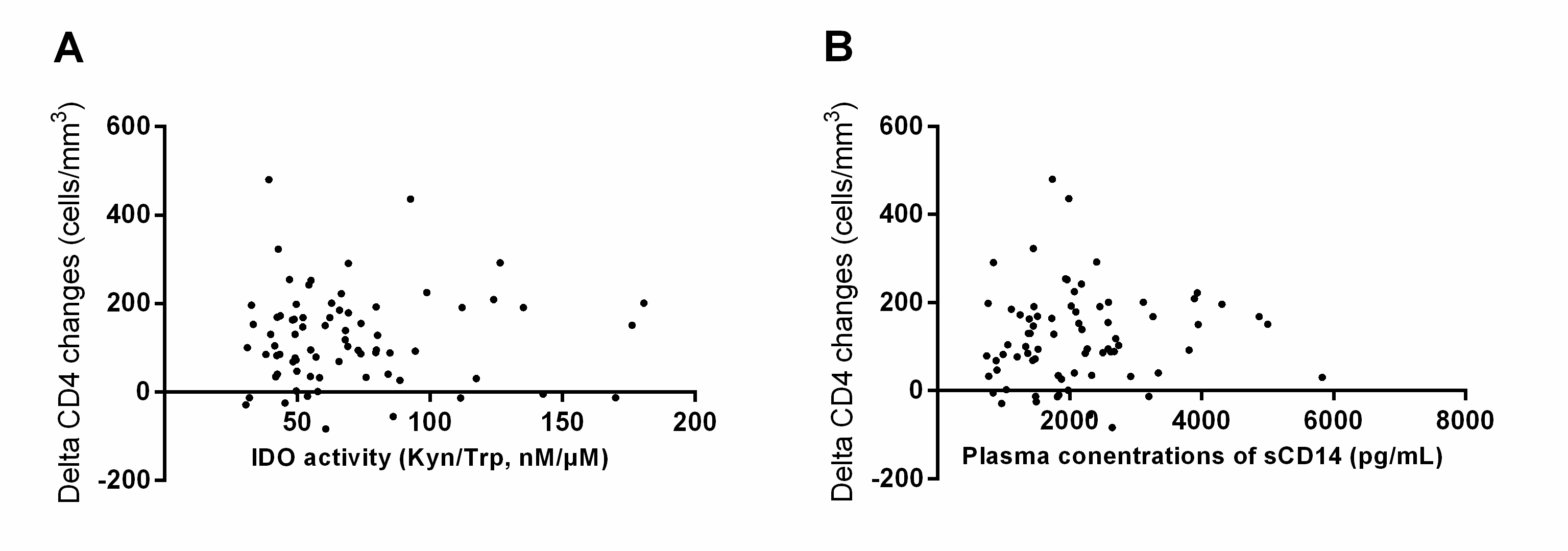

Supplement: Figure S2 — The relationship between delta CD4+ T cell changes from baseline to IDO activity (A) and plasma sCD14 level (B) in HIV-infected patients. (TIF) [file pone.0100446.s002.tif]
